# Monitoring of polycyclic aromatic hydrocarbons and probabilistic health risk assessment in yogurt and butter in Iran

**DOI:** 10.1002/fsn3.2180

**Published:** 2021-02-14

**Authors:** Amin Kiani, Mahsa Ahmadloo, Mojtaba Moazzen, Nabi Shariatifar, Saeed Shahsavari, Majid Arabameri, Mohammad Mahdi Hasani, Ali Azari, Mosaad A. Abdel‐Wahhab

**Affiliations:** ^1^ Department of Public Health School of Public Health Fasa University of Medical Sciences Fasa Iran; ^2^ Department of Food Safety and Hygiene School of Public Health Qazvin University of Medical Sciences Qazvin Iran; ^3^ Department of Environmental Health Engineering School of Public Health Tehran University of Medical Sciences Tehran Iran; ^4^ Health Products Safety Research Center Qazvin University of Medical Sciences Qazvin Iran; ^5^ Food Safety Research Center (salt) Semnan University of Medical Sciences Semnan Iran; ^6^ Department of Environmental Health Engineering Faculty of Health Tehran Medical Sciences Islamic Azad University Tehran Iran; ^7^ Department of Environmental Health Engineering Faculty of Health Kashan University of Medical Sciences Kashan Iran; ^8^ Food Toxicology & Contaminants Department National Research Center Dokki Egypt

**Keywords:** butter, gas chromatography‐mass spectrometry (GC/MS), health risk assessment, occurrence, polycyclic aromatic hydrocarbons, yogurt

## Abstract

This study was conducted to determine the polycyclic aromatic hydrocarbons (PAHs) levels and health risk of yogurt and butter samples collected from Tehran using MSPE/GC‐MS (magnetic solid‐phase extraction/gas chromatography‐mass spectrometry). The results revealed that the limit of detection (LOD) and limit of quantification (LOQ) were ranged from 0.040 to 0.060 and 0.121 to 0.181 μg/kg, respectively; with recoveries ranged from 86.1% to 100.3%. The highest mean of total PAHs was higher in butter (6.87 ± 1.21 μg/kg) than in yogurt (3.82 ± 0.54 μg/kg). The level of benzo (a)pyrene in all samples was lower than of standard levels of the European Union (EU). The highest value of all PAHs in samples was recorded in the winter season and also in the expiration date. The percentile 95% of the total hazard quotient (THQ) due to the consumption of yogurt and butter recorded 1.33E‐02 and 3.69E‐04 in adults and 6.12E‐02 and 1.75E‐03 in children, respectively. The percentile of 95% incremental lifetime of cancer risk (ILCR) due to the ingestion of yogurt and butter recorded 1.17E‐06 and 2.02E‐08 for adults and 5.51E‐06 and 9.46E‐08 for children, respectively. The rank order of 7 PAHs in adult and children based on P95% Hazard Quotient (HQ) in all samples was benzo(a)anthracene (BaA) > pyrene (P) > fluorene (F) > fluoranthene (Fl) > acenaphthylene (Ace) > anthracene (A) > naphthalene (NA). According to the Monte Carlo Simulation (MCS) method, health‐risk assessment showed that children and adults are not at significant health risk.

## Graphical abstract

Determine the polycyclic aromatic hydrocarbons (PAHs) levels and health risk of yogurt and butter samples collected from Tehran using MSPE/GC‐MS. The highest mean of total PAHs was higher in butter (6.87 ± 1.21) than in yogurt (3.82 ± 0.54). The rank order of 7 PAHs in adult and children based on P95% Hazard Quotient (HQ) in all samples was BaA > PY > F > FL > AcP > A > NA.

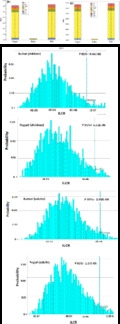

## 1 INTRODUCTION

Nowadays, several dangerous and carcinogenic compounds are detected in food and environment, one of which is PAH which are found in air, water, and food (Alharbi et al., 2018; Ali & Aboul‐Enein, 2005; Ali et al., 2003, 2005, 2011, 2018; Basheer, 2018). In some industrial areas, the high levels of PAH in unprocessed foods suggest a contamination due to the transport of contaminated particles and natural emissions from forest and volcanic fires or the burning of oil reservoirs (Orecchio et al., 2009; Rey‐Salgueiro et al., 2009). There is a positive correlation between the exposure to PAHs and the occurrence of several diseases such as stomach cancer, alteration in the lung including DNA adduct, biochemical and cytogenetic alterations (Rawash et al., 2018; Santonicola et al., 2017).

The United States Environmental Protection Agency (USEPA) has involved 16 of these persistent compounds in its list of persistent organic pollutants (Orecchio et al., 2009; Suchanová et al., 2008). The International Agency for Research on Cancer (LARC) classified BaP as category 2A (probably carcinogenic) to humans (N Aguinaga et al., 2008). Therefore, the EU established a limit of less than 1 µg/kg of BaP for the processed cereal‐based foods prepared for young children, infants, and baby foods (EC 2005).

People can be exposed to PAHs through three main routes including respiratory, skin, and foods (Chung et al., 2010; Cirillo et al., 2004; Shariatifar et al., 2020), and they were reported to be accumulated in various types of food matrices including dairy products (milk, yogurt, butter), etc. (Chung et al., 2010; Cirillo et al., 2004; Shariatifar et al., 2020). These PAHs are persistent in various food products during cooking procedures, thermal processes (such as baking, smoking, grilling, frying), processing, and packaging (Farhadian et al., 2010; Gorji et al., 2016).

Milk and milk products are essential for infants, children and adults, due to their high content of macro and micronutrients (Han et al., 2014; Iwegbue et al., 2014), and yogurt and butter are playing an important role in the human diet (Lee et al., 2015; Martorell et al., 2010). Therefore, milk and dairy products may contain PAHs due to the lipophilic property of PAHs even after exposure to various heat treatments (Lin et al., 2016).

Several methods were developed for the determination of PAHs in milk and dairy product including Liquid–liquid extraction (LLE), Soxhlet apparatus, Solid‐phase extraction (SPE), and Pressurized liquid extraction (PLE). The most widely used methods for the extraction of PAHs from an intricutionate sol matrix includes silica cartridges, LLE after saponification with caustic soda, straight immersion solid‐phase microextraction (SPME), columns with gel beads, and 2 phase hollow liquid phase microextraction (HF‐LPME) (Basheer & Ali, 2018; Moazzen, Khaneghah, et al., 2018; Moazzen, Mahvi, et al., 2018). However, due to the fat content and other materials, alcoholic or caustic solutions are used for the saponification to avoid difficulties from lipids followed by the use of SPE or LLP for the extraction (Moazzen et al., 2013; Naccari et al., 2011). Moreover, several methods were established for the determination of PAHs in milk and dairy products including gas chromatography with flame ionization detection (GC/FID) or gas chromatography with mass detection (GC/MS), high‐performance liquid chromatography (HPLC) equipped with ultraviolet and fluorescence detection (UV/FL) and fluorescence detector (FLD) (Ali et al., 2019; Grova et al., 2002; Kamankesh et al., 2015).

For the toxicological evaluation of chemical mixes, toxic equivalency (TEQ) is a concept to indicate the whole toxicity of a mixture, like PAHs. The TEQ was designed to estimate the toxicity and hazards of toxic chemicals related to conventional toxic compounds. However, the toxic equivalency factor (TEF) of each compound shows the level of toxicity compared to the alternative composition, which gives the reference value 1. This TEF method is a common technique to evaluate the risk of PAHs and it was used by USEPA and WHO (1998). The final toxic point and the toxicity grade are the main factors for the TEQ method and TEFs of PAHs, which were proposed based on the growth in the incidence of tumors reported in animal study; however, TEFs for each PAHs compounds were based on their cancer outcomes of various carcinogenic researches (Yoon et al., 2007).

Considering the relatively high consumption of milk products in the Iranian basket, severe air pollution and partly water pollution in a densely populated city like Tehran, and also lack of coherent research about PAHs in dairy product with risk assessment in Iran, it is important to evaluate the amount of PAHs in milk products. Therefore, the goals of the present study are the determination of PAHs in some milk products (butter and yogurt) with the MSPE‐GC/MS method and assessment of the potential health risk produced by PAHs intake using the cancer potency of BaP as a reference member.

## 2 MATERIALS AND METHODS

### 2.1 Sampling

In this study, 96 samples of milk products (48 yogurt and 48 butter samples) were purchased from local markets in different regions of Tehran, Iran in May, August, December 2018, and February 2019. Three different brand sources were purchased for each type of milk products. All samples were completely homogenized and then five grams of each sample were kept in airtight dishes at −20°C until analysis.

### 2.2 Standards and reagents

Standards PAH mix, including 16 mentioned PAHs (benzo(a)pyrene [BaP], acenaphthylene [Ace], naphthalene [NA], fluorene [F], acenaphthene [Ac], phenanthrene [Pa], fluoranthene [Fl], anthracene [A], benzo(a)anthracene [BaA], pyrene [P], benzo(b)fluoranthene [BbF], chrysene [Ch], benzo(k)fluoranthene [BkF], dibenzo[a,h]anthracene [DhA], indeno[1,2,3‐cd]pyrene [IP] and benzo[g,h,i]perylene [BgP]) was purchased from Supelco (Bellefonte, PA, U. S.). The standard solutions were prepared in dichloromethane, with all mentioned PAHs concentration of 0.1 mg/ml. Stock standard solutions were mixed with methanol‐dichloromethane (50:50, v/v) every week for the preparation of a working mixed solution (1 µg/ml for each mentioned PAHs) which was used to measure the extraction performance under various conditions. The working and stock solutions were preserved at 4°C and biphenyl was used as an internal standard at a concentration of 0.05 µg/ml in methanol. Multi‐walled carbon nanotubes were purchased from Hanwha Nanotech (MWCNT CM‐95, Korea) and the adsorbent of MWCNT‐MNP was prepared as described previously (Rastkari & Ahmadkhaniha, 2013). All other chemicals and solvents were of analytical grade.

### 2.3 Samples preparation

#### 2.3.1 Yogurt

Yogurt samples were prepared as described in our previous work (Moazzen et al., 2013). This method includes 3 major parts; (a) sample cleanup: Five grams of the sample was ground to fine particles with a pulverization then mixed with 1 ml internal standard and the mixture was homogenized for 15 min. The extraction solution containing 7.5 ml acetonitrile‐methanol (70%: v/v) and 7.5 ml potassium hydroxide (1 M) was added to the homogenized mixture then was mixed and sonicated for 7 min in a bath of ultrasonic at 50°C. The mixture was centrifuged for 10 min at 8,944 × g, the fat was removed from the mixture in the lipid freezing filtration method (Ahmadkhaniha et al., 2009) and the pH was adjusted to 6.5 using HCl (1 M); (b) Analytes adsorption: after a rudimentary cleanup, the aqueous phase was transferred to another container, 500 mg NaCl and 10 mg of synthesized multi‐walled carbon nanotubes magnetic nanoparticle (MWCNT‐MNP) as adsorbent was added to the mixture and vortex‐mixed vigorously for 5 min. The magnetic synthesized adsorbent was collected to the side of the vial by an exterior magnet; (c) Analytes desorption from the magnetic adsorbent: dichloromethane (5 ml) was added to the elute of PAHs compounds from the synthesized adsorbent, mixed with strong vortex‐mixing for 3 min and the supernatant was discarded. The magnetic adsorbent was collected by an exterior magnet to the side of the vial. This phase was repeated for another one more time, afterward, the solvent was evaporated to dryness at 30°C using a gentle stream of pure nitrogen gas. Then the extract was re‐dissolved in 50 µl acetonitrile‐methanol (50:50 v/v), vigorously shaken for 1 min with the vortex‐mixer, and 1 µl was injected into the GC/MS device. The outcomes of the optimization experiments showed that the mentioned technique permitted for reproducible, quantitative extraction of polycyclic aromatic hydrocarbons (Moazzen et al., 2013). The blank samples include internal standard and QC (quality control) were examined at the beginning of the analysis, middle, and end of each sample. The mean values of results were used for quantification and all the samples in this research were examined in duplicate.

#### 2.3.2 Butter

Due to the high‐fat content in butter, the samples were homogenized before extraction. Five grams of butter were subjected to saponification with methanolic Potassium hydroxide, cold extracted with cyclohexane‐dimethylformamide, and cyclohexane for re‐extraction. The clean‐up technique for the extracted sample was carried out by gel permeation chromatography with Bio‐Beads SX‐3 column followed by adsorption chromatography on silica gel. The final phase was the reduction of the cleaned extracts to a suitable analytical volume using acetonitrile as a solvent (Falco et al., 2003).

### 2.4 Analytical conditions and instrumentals

The analysis was conducted on a GC device of the Agilent 6890 with an MS detector 5973 quadrupoles (Agilent Technologies). The GC was adjusted with a capillary column of DB‐5 ms (30 m, 0.25 mm i.d., 0.25 µm film thickness) and the mode of splitless was for the inlet. The temperatures were adjusted as follows: 290°C for injector; the initial temperature of the oven was 70°C, kept for one min and increased to 295°C at a rate of 10°C/min then kept for seven min. The transfer line temperature was kept at 300°C and the constant flow of helium as a carrier gas was 1 ml/min. The quadrupole temperature was kept at 150°C and source temperatures were retained at 230°C. The energy of the electronic beam of the MS was adjusted at 70 eV. The identification was carried out by comparing the obtained mass spectra and times of retention to reference spectra and times of retention that were obtained by injection of the calibration standards in the same conditions of GC/MS. The spike calibration standard approach to overcome the problems caused by the matrix was used. In this approach, calibration standards are ready by the addition of standards solution to samples that are subjected to the same sample preparation procedure which is intended to be used for unknown samples. The PAHs analytes were quantified using SIM mode and each sample was injected in duplicate.

### 2.5 Health risk assessment

To evaluate the carcinogenic risk of PAHs, the toxicity equivalency factors (TEFs) of benzo(a)pyrene (TEQ BaP) were used. The estimation of TEQ BaP was carried out using a TEFs (Saito et al., 2014). The TEF fixed as one for BaP and dibenzo[a,h]anthracene (DhA); 0.001 for each of naphthalene (NA), acenaphthylene (Ace), acenaphthene (Ac), fluorine (F), Phenanthrene (Pa), fluoranthene (F) and pyrene (P); 0.01 for each of anthracene (A), chrysene (Ch) and benzo[g,h,i]perylene (BgP) and 0.1 for each of benzo(a)anthracene (BaA), benzo(b)fluoranthene (BbF), benzo(k)fluoranthene (BkF), indeno [1,2,3‐cd]pyrene (IP), benzo(a)pyrene (BaP) and dibenzo[a,h]anthracene (DhA). TEQ BaP for dietetic contact to PAHs in milk products was computed matched to the following equation (EPA (2010)

(1) $${\mathrm{TEQ}}_{\mathrm{BaP}}=\sum_{i=1}^{n}C_{i}\times {\mathrm{TEF}}_{i}$$

Where C is a quantitative analysis of PAHs and the TEFi is the toxicity equivalency factor of biotype (i) in milk products.

To estimate the hazard of noncarcinogenic of PAH compounds in milk products, the average daily dose (ADD) was applied and calculated by the following equation:

(2) $$\mathrm{ADD}=\frac{C\times \mathrm{IR}}{\mathrm{BW}}$$

Due to the carcinogenic hazard, the chronic daily intake (CDI) was calculated according to the following equation:

(3) $$\mathrm{CDI}=\frac{C\times \mathrm{IR}\times \mathrm{ED}\times \mathrm{EF}}{\mathrm{BW}\times \mathrm{AT}}$$

This study assumed that milk items consumed 365 d/year, C is the PAHs levels in milk (mg/kg); IRi as the daily intake of the milk products was set as 73 g/day and 2 g/day for yogurt and butter, respectively (Abdollahi et al., 2014); ED is the time‐period of PAHs exposure for adults and children (70 and 6 years old, respectively) as suggested by (Karami et al., 2020). EF is the frequency exposure of PAHs (350 days/years) (Jahanbakhsh et al., 2019); the average body weight (BW) is 15 kg for children and 70 kg for an adult was used in the calculation of PAHs risk assessment (Samiee et al., 2020) and AT is the average time for an adult is 25,550 days and for the children 2,190 days.

The hazard of noncarcinogenic was calculated based on Equation 4:

(4) $$\mathrm{HQ}=\frac{\mathrm{ADD}}{\mathrm{RfD}}$$

Where, HQ is the hazard quotient; ADD is the average daily dose (mg/kg/d); RfD is the oral reference dose (mg/kg/d) as suggested by (USEPA, 2017) is NA (0.02), AcP (0.06), FL (0.04), AN (0.3), FLUR (0.04), PY (0.03) and BaA (0.0003). If THQ > 1 value, the exposed population is considered at health risk, but if THQ ≤ 1, the health risk is not likely (Ghasemidehkordi et al., 2018). In considering the effects of PAHs on the risk of noncarcinogenic, the total hazard quotient (THQ) was determined by the Equation below:

(5) $$\mathrm{THQ}=\sum {\mathrm{HQ}}_{1}\cdots {\mathrm{HQ}}_{n}$$

However, the ILCR of BaP of the milk products was determined according to the following Equation (Fakhri et al., 2018).

(6) $$\mathrm{ILCR}={\mathrm{CDI}}_{\mathrm{BaP}}\times {\mathrm{CSF}}_{\mathrm{BaP}}$$

Where: ILCR is incremental lifetime cancer risk and CDI is the chronic daily intake of BaP (mg/kg/d); CSF is the cancer slope factor of BaP which is equal to 7.3 mg/kg/d (Huang et al., 2016; Samiee et al., 2020; USEPA, 2016). According to USEPA, cancer is negligible when ILCR < 10–6, risk, but if ILCR > 10–4, the risk of cancer is unacceptable and finally, when ILCR is between 10–6 to 10–4, the risk of cancer is tolerable for consumers (Huang et al., 2016; USEPA, 2016).

The incremental lifetime carcinogenic risk (ILCR) was evaluated and noncarcinogenic (HQ) of the milk product content of PAHs was navigated by MCS (Crystal Ball v 11.1.2.4.600 software, Oracle, Decisioneering, Denver, CO, USA). To increase the accuracy of health‐risk assessment by considering uncertainties, USEPA presented the MCS method (EPA, 2016). The simulation executed by the presented factors, and the MCS model was shown for 10,000 repetitions. Eventually, the mean and 95th percentile of the ILCR and HQ distribution was selected to assess if the exposed society is at risk or not. For estimation of carcinogenic risk of PAHs in milk products, actual ILCR was calculated according to the following equation:

(7) $${\mathrm{ILCR}}_{\left(\mathrm{act}\right)}=P95\%I{\mathrm{LCR}}_{\mathrm{Yo}}+P95\%I{\mathrm{LCR}}_{\mathrm{Bu}}$$

Where: ILCR (act) is actual ILCR; ILCR_yo_ is ILCR for yogurt and ILCR_bu_ and is ILCR for butter.

For estimation of actual noncarcinogenic risk of PAHs in milk products, actual THQ was calculated based on the following equation:

(8) $${\mathrm{THQ}}_{\left(\mathrm{act}\right)}=P95\%{\mathrm{THQ}}_{\mathrm{Yo}}+P95\%{\mathrm{THQ}}_{\mathrm{Bu}}$$

Where THQ (act) is actual THQ; HQbu, is the HQ for butter and HQyo, is the HQ for yogurt.

### 2.6 Statistical examination

The results were presented as mean ± *SD* and the statistical significance was carried out by SPSS (version 24.0) software. Data for PAHs levels in milk products (yogurt and butter) were evaluated for normality (Kolmogorov‐Smirnov test). For normally distributed data, comparisons among yogurt and butter were assessed using an independent *t*‐test also if non‐normally distributed data, comparisons among yogurt and butter were assessed using Mann–Whitney as shown in Table 1. Values were considered statistically significant when *p* <.05. In cases, when PAH analytes were ND (not detected), 1/2 of the LOD was used to calculate the mean concentration.

**TABLE 1:** Linear range (µg/kg), Limit of detection (LOD; µg/kg), limit of quantification (LOQ; µg/kg), Coefficient of estimation (r2), repeatability relative standard deviation (RSDr; *n* = 6), and reproducibility relative standard deviation (RSDR; *n* = 6)

Target compound	Linear range (µg/kg)	Limit of detection (LOD) (µg/kg)	Limit of quantification (LOQ) (µg/kg)	Coefficient of estimation (r2)	Recoveries (%)	Repeatability (RSDr) (%)	Reproducibility (RSDR) (%)
NA	0.050–1.000	0.040	0.121	0.988	91.2	9	6, 10, 14
Ace	0.050–1.000	0.040	0.121	0.981	93.4	10.1	9, 12, 17
Ac	0.050–1.000	0.040	0.121	0.990	86.1	3.2	5, 10,12
F	0.050–1.000	0.040	0.121	0.992	96.1	6.4	14, 8, 16
Pa	0.050–1.000	0.050	0.150	0.991	99.7	7	13, 11, 8
A	0.050–1.000	0.040	0.121	0.989	89.9	10	7, 12, 10
Fl	0.050–1.000	0.040	0.121	0.983	100.1	9	10, 13, 7
P	0.050–1.000	0.055	0.165	0.986	93.5	9.1	11, 9, 18
BaA	0.050–1.000	0.040	0.121	0.984	94.7	5	6, 7, 12
Ch	0.050–1.000	0.040	0.121	0.991	98.1	4.5	9, 13, 11
BbF	0.050–1.000	0.060	0.181	0.992	99.9	8	12, 7, 10
BkF	0.050–1.000	0.040	0.121	0.986	100.3	9	14, 11, 8
BaP	0.050–1.000	0.040	0.121	0.988	94.5	8.6	7, 15, 11
IP	0.050–1.000	0.040	0.121	0.989	89.9	6	5, 9, 12
DhA	0.050–1.000	0.040	0.121	0.992	97.1	7	14, 6, 10
BgP	0.050–1.000	0.040	0.121	0.991	97.8	9.2	7, 5, 12

Note

RSDR of 1 µg/Kg, 5 µg/Kg, and 10 µg/Kg standard value (*n* = 6).

## 3 RESULTS AND DISCUSSIONS

### 3.1 Performance evaluation of the analytical method

The extracted PAHs were then determined using a GC‐MS technique. Toward the recognition goal, the full scan mass spectrum, 4 characteristic ions ratios, and the RTT of ± 0.5% tolerance criteria were applied for the quantification purpose compared to the standard, and the most intense ions were used for each compound and these compounds were then quantified using SIM (selected ion monitoring) mode. According to Moazzen et al. (2013), one quantitation and two qualifier ions were controlled for each compound. The optimum conditions for the analysis were used for the establishment of the calibration curves (0.050–1.000 µg/kg) considering the coefficient of correlation of 0.981–0.992. The LOQs and LODs of PAHs compounds were 0.121–0.181 µg/kg and 0.040–0.060 µg/kg, respectively. The accuracy of the technique was assessed according to interday precision via the QC analysis for samples ready at four three repeated days. Additionally, the values of interday precision for all PAHs compounds were less than 8.7% and the recorded values were 3.2% to 10.1% for repeatability and 5%–18% for reproducibility with estimated recoveries of 86.1%–100.3% (Table 1). The feasibility and reliability of this method were also confirmed by the measurement PAHs in the milk, doogh, and milk powder (Kiani et al., 2019).

### 3.2 Comparing the mean values of PAHs in the tested milk products

The mean values of PAHs compounds based on the type of dairy products are presented in Table 2 and showed that the highest levels of all PAHs compounds were found in the butter. The recorded means of total PAHs in yogurt and butter were 3.82 ± 0.54 μg/kg and 6.87 ± 1.21 μg/kg, respectively (Table 2). The results also indicated that the higher the amount of fat content and the lower the amount of water content, the more contaminant of PAHs was observed. In this study, P recorded the maximum mean value in yogurt (1.39 ± 0.10 μg/kg) and butter (2.45 ± 0.38 μg/kg). Among the groups suggested in EC Regulation Number 835/ 2011 (2011), a maximum value of PAH4 of 1 µg/kg was reported only in milk powder which corresponding to the infant products. The mean levels of PAH4 observed in the present study were below the standard limits since the recorded values were 0.23 ± 0.08 and 0.60 ± 0.15 μg/kg for yogurt and butter, respectively. The EC set 1 μg/kg as a maximum level for BaP in the baby foods for infant and young children, processed cereal‐based foods, infant milk, and infant formulae. The current results showed that the levels of BaP were also below the standard levels (between 0.02–0.08 μg/kg) in all samples and the recorded means were 0.03 ± 0.02 and 0.03 ± 0.01 µg/kg in butter and yogurt, respectively. In a previous study, we reported that the means of total PAHs, PAH4, and BaP in doogh samples were 1.96 ± 0.39, 0.15 ± 0.07, and 0.03 ± 0.01 µg/kg, respectively (Kiani et al., 2019). In this concern, Falco et al. (2003) reported a mean concentration of 6.636 µg/kg for total PAHs in the dairy product (yogurt and cheese) and it was reported that the detection range of the sum of PAHs in conventional and organic yogurt ranged between 38.28 and 306.59 µg/kg fat (Rodríguez‐Hernández et al., 2015). Recently, Kacmaz (2019) reported that the highest concentrations for the sum of the 4PAHs (benz[a]anthracene, chrysene, benzo[b]fluoranthene and benzo[a]pyrene) were 0.33 ± 0.18 μg/kg of retail Turkish yogurt, 0.59 μg/kg and 0.95 μg/kg in yogurt with low‐fat and high‐fat content, respectively (Kacmaz 2019). Additionally, previous studies revealed that the concentrations of total PAHs were 3.695 ± 0.43 – 5.443 ± 0.66 µg/g in milk and dairy‐based products (E.‐S. A. Rawash et al., 2018) and 72.8 µg/kg in butter samples collected from Egypt (Loutfy et al., 2007), 2.4 to 4 μg/kg in Finnish butter (Hopia et al., 1986), 0.65 and 18.7 μg/kg in fresh Spanish yogurt and butter, respectively (Martorell et al., 2010) and 0.61 and 18.3 μg/kg in fresh yogurt and butter from Northwest Spain, respectively (Martí‐Cid et al., 2008).

**TABLE 2:** Mean ± *SD* of PAH factors in yogurt and butter in Iran (μg/kg)

	Type of dairy products								
	Yogurt				Butter				
	*N*	Min	Max	Mean ± *SD*	*N*	Min	Max	Mean ± *SD*	
NA	48	0.02	0.02	0.02 ± 0.00	48	0.02	0.02	0.02 ± 0.00	—
Ace	48	0.30	1.17	0.80 ± 0.27	48	1.10	2.30	1.62 ± 0.32	*p* <.001
Ac	48	0.02	0.02	0.02 ± 0.00	48	0.02	0.02	0.02 ± 0.00	—
F	48	0.02	0.02	0.02 ± 0.00	48	0.02	0.02	0.02 ± 0.00	—
Pa	48	1.06	1.42	1.21 ± 0.12	48	1.40	2.80	2.01 ± 0.38	*p* <.001
A	48	0.02	0.02	0.02 ± 0.00	48	0.02	0.02	0.02 ± 0.00	—
Fl	48	0.02	0.02	0.02 ± 0.00	48	0.02	0.02	0.02 ± 0.00	—
P	48	1.28	1.64	1.39 ± 0.10	48	1.90	3.20	2.45 ± 0.38	*p* <.001
BaA	48	0.02	0.02	0.02 ± 0.00	48	0.02	0.02	0.02 ± 0.00	—
Ch	48	0.02	0.12	0.05 ± 0.03	48	0.02	0.12	0.05 ± 0.03	1.000
BbF	48	0.08	0.23	0.15 ± 0.05	48	0.37	0.75	0.51 ± 0.11	*p* <.001
BkF	48	0.02	0.02	0.02 ± 0.00	48	0.02	0.02	0.02 ± 0.00	—
BaP	48	0.02	0.02	0.02 ± 0.00	48	0.02	0.02	0.02 ± 0.00	.737
IP	48	0.02	0.02	0.02 ± 0.00	48	0.02	0.02	0.02 ± 0.00	—
DhA	48	0.02	0.02	0.02 ± 0.00	48	0.02	0.02	0.02 ± 0.00	—
BgP	48	0.02	0.06	0.03 ± 0.01	48	0.02	0.08	0.03 ± 0.02	.087
Total	48	3.01	4.86	3.82 ± 0.54	48	5.01	9.47	6.87 ± 1.21	*p* <.001
PAH4	48	0.14	0.41	0.23 ± 0.08	48	0.43	0.93	0.60 ± 0.15	*p* <.001

Due to this concern, many studies have been conducted on other food samples in different countries (Table 3) revealed shown that the results are varied due to different types of food. The reasons for these variations can be briefly summarized as follow: the low level may be due to the remoteness of a farm from the industrial environment, cities, roads, and do not consume or reduce the use of fossil fuels in livestock buildings to heat the environment, the use of healthy food and non‐polluting water for animals, less food fats, the use of correct methods in food processing such as using less heat, steaming and not contacting foods with very hot surfaces and direct flame. However, the high levels can be due to the failure to comply with the above which increased the PAHs contaminant in food types (Kishikawa et al., 2003; Lawrence & Weber, 1984; Lin et al., 2016; Rawash et al., 2018; Rey‐Salgueiro et al., 2009; Santonicola et al., 2017; Yurchenko & Mölder, 2005).

**TABLE 3:** Comparison of PAHs contamination levels in different study

Location of study	Analyte	Sample type	Amount	Detection method	References
France	8 PAHs	Fresh milk	Ʃ PAHs (26.7 ± 10.8 ng/g)	GC‐MS	(Grova et al., 2002)
Spain	16 PAHs	Infant formula	ND	GC‐MS	(Aguinaga et al., 2007)
		Half‐fat milk	ND		
		Skimmed milk	ND		
		Full fat milk	Ʃ FLUR and PY (2.16 ± 0.43 µg/L)		
Spain	10 PAHs	Infant formula	ND	GC‐MS	(Aguinaga et al., 2008)
		Soya milk	ND		
		Full‐fat milk	ND		
		Skimmed milk	Ʃ AcPY, FL, PHEN, AN, FLUR and PY (449.4 ± 31.51 ng/L)		
Italy	16 PAHs	Milk	ND	GC‐MS	(Bianchi et al., 2008)
Taiwan	16 PAHs	Milk	NaP, AcP, Flu, Pa and in some samples Bap	GC‐MS	(Chung et al., 2010)
Nigeria	16 PAHs	Infant formula:	Ʃ PAHs	GC‐MS	(Iwegbue et al., 2014)
		0–6 months	0.1 to 1.98		
		6–12 months	0.05 to 1.98		
		1–3 years	0.02 to 2.54		
		0–12 months	0.51 to 0.7 µg/kg		
Egypt	15 PAHs		Ʃ PAHs	GC‐MS	(Abou‐Arab et al., 2014)
		Farm raw milk	1.01 µg/kg		
		Commercial raw milk	0.37 µg/kg		
		Pasteurized milk	0.2 µg/kg		
Mexico	16 PAHs	Milk	Ʃ PAHs	GC‐FID	(Gutiérrez et al., 2015)
		2008	2.06 ± 2.83		
		2009	1.65 to 2.14		
		2010	1.24 ± 1.28 µg/g		
Korea	8 PAHs		Ʃ PAHs	GC‐MS	(Lee et al., 2015)
		Dairy products	1.52 µg/kg		
		Sea foods	1.06 µg/kg		
Romania	15 PAHs	Milk powders	Ʃ PAHs (0.47 ± 0.04 to 1.4 ± 0.17 µg/kg)	GC‐MS	(Dobrinas et al., 2016)
China	6 PAHs	Milk	Ʃ PHE, ANT, FLA and PYR (6.0 ng/g)	GC‐MS	(Lin et al., 2016)
Canada	15 PAHs		Ʃ PAHs	HPLC‐FID	(Lawrence & Weber, 1984)
		Skim Milk	0–2.7 µg/kg		
		Infant formula	8.1 µg/kg		
Japan	12 PAHs		Ʃ PAHs	HPLC‐FID	(Kishikawa et al., 2003)
		Commercial milk	0.99 ± 0.37 µg/kg		
		Infant formula	2.01 ± 0.30 µg/kg		
		Human milk	0.75 ± 0.47 µg/kg		
Poland	16 PAHs	Milk powder	Ʃ BaA, Ch, BbF, BkF, BaP and BP(1.83 µg/kg)	HPLC‐FID	(Wegrzyn et al., 2006)
Czech republic	15 PAHs	Industrial smoked cheese	Ʃ PAHs (0.11 µg/kg)	HPLC‐FID	(Suchanová et al., 2008)
Spain	11 PAHs	Infant food	BkF (0.1–0.3 µg/kg)	HPLC‐FID	(Rey‐Salgueiro et al., 2009)
Poland	19 PAHs	Infant formula	Ʃ PAHs (0.28–7.45 µg/kg)	HPLC‐FID/DAD	(Ciecierska & Obiedziński, 2010)
Calabria	16 PAHs		Ʃ PAHs	HPLC‐FID	(Naccari et al., 2011)
		Raw milk	5.44 ng/g		
		Pasteurized milk	6.52 ng/g		
		Semi‐skimmed milk	5.94 ng/g		
		Whole milk	7.75 ng/g		
Italy	16 PAHs		Ʃ PAHs	HPLC‐UV	(Cirillo et al., 2004)
		Non‐smoked Cheese	59.11–160.05 µg/kg		
		Smoked cheese	67.49–399.90 µg/kg		
Korea	7 PAHs		Ʃ PAHs	HPLC‐FID	(Cho & Shin, 2012)
		Infant formula	0.43 µg/kg		
		Mixed milk powders	0.46 µg/kg		
Argentina, Brazil	15 PAHs	Milk powders	Ʃ PAHs (11.8–78.4 µg/kg)	HPLC‐FID	(Garcia Londoño et al., 2013)
Korea	8 PAHs	Infant formula	Ʃ PAHs (0.09–0.18 µg/kg)	HPLC‐FID	(Han et al., 2014)
Iran	15 PAHs	Kebab (grilled meat)	Ʃ PAHs (7.37–17.94 µg/kg)	GC‐MS	(Gorji et al., 2016)
Iran	3 PAHs	Grilled meat	Ʃ PAHs (3.51–132 ng/g)	HPLC‐FD	(Farhadian et al., 2010)
Sweden	1 PAHs	Swedish smoked meat	Ʃ BaP (6.6–36.9 µg/kg)	HRGC–MS	(Wretling et al., 2010)
Estonia	6 PAHs	smoked fish	Ʃ PAHs (12.37 µg/kg)	GC‐MS	(Yurchenko & Mölder, 2005)
Spain	16 PAHs		Ʃ PAHs	HRGC‐ HRMS	(Martorell et al., 2010)
		Meat and meat products	38.99 μg/kg		
		Fish and shellfish	2.87 μg/kg		
		Vegetables	1.22 μg/kg		
		Fruits	0.81 μg/kg		
		Milk	0.47 μg/kg		
		Oils and fats	18.75 μg/kg		
Italy	22 PAHs	Coffee	Ʃ PAHs 0.52 to 1.8 µg/L	GC‐MS	(Orecchio et al., 2009)

The means of the PAHs based on the type of milk product in different seasons are presented in Table 4 and indicated that the means of all PAH compounds recorded the highest value in the winter samples. The maximum means of total PAHs and PAH4 were 8.13 ± 1.02 and 0.55 ± 0.09 μg/kg in butter, 4.40 ± 0.34 and 0.32 ± 0.06 μg/kg in yogurt. The current results also showed that the maximum BaP mean in all of the brands was below the standard levels of the EU. The lowest mean recorded for total PAHs and PAH4 were in spring, which reflects the high pollutants in winter due to the use of fossil fuels in livestock, or water, air, and livestock feed due to the lack of fresh fodder in this season.

**TABLE 4:** Mean ± *SD* of PAH factors in yogurt and butter in Iran based on seasons of year (μg/kg)

Type of dairy products								
Analyte	Yogurt				Butter			
	Season				Season			
	Spring	Summer	Autumn	Winter	Spring	Summer	Autumn	Winter
	Mean ± *SD*	Mean ± *SD*	Mean ± *SD*	Mean ± *SD*	Mean ± *SD*	Mean ± *SD*	Mean ± *SD*	Mean ± *SD*
NA	0.02 ± 0.00	0.02 ± 0.00	0.02 ± 0.00	0.02 ± 0.00	0.02 ± 0.00	0.02 ± 0.00	0.02 ± 0.00	0.02 ± 0.00
Ace	0.52 ± 0.21	0.72 ± 0.17	0.91 ± 0.19	1.07 ± 0.08	1.33 ± 0.16	1.43 ± 0.19	1.80 ± 0.23	1.90 ± 0.28
Ac	0.02 ± 0.00	0.02 ± 0.00	0.02 ± 0.00	0.02 ± 0.00	0.02 ± 0.00	0.02 ± 0.00	0.02 ± 0.00	0.02 ± 0.00
F	0.02 ± 0.00	0.02 ± 0.00	0.02 ± 0.00	0.02 ± 0.00	0.02 ± 0.00	0.02 ± 0.00	0.02 ± 0.00	0.02 ± 0.00
Pa	1.10 ± 0.03	1.14 ± 0.05	1.28 ± 0.10	1.32 ± 0.09	1.60 ± 0.13	1.87 ± 0.10	2.17 ± 0.29	2.40 ± 0.32
A	0.02 ± 0.00	0.02 ± 0.00	0.02 ± 0.00	0.02 ± 0.00	0.02 ± 0.00	0.02 ± 0.00	0.02 ± 0.00	0.02 ± 0.00
Fl	0.02 ± 0.00	0.02 ± 0.00	0.02 ± 0.00	0.02 ± 0.00	0.02 ± 0.00	0.02 ± 0.00	0.02 ± 0.00	0.02 ± 0.00
P	1.32 ± 0.07	1.34 ± 0.04	1.42 ± 0.09	1.49 ± 0.10	2.03 ± 0.12	2.23 ± 0.12	2.68 ± 0.19	2.87 ± 0.26
BaA	0.02 ± 0.00	0.02 ± 0.00	0.02 ± 0.00	0.02 ± 0.00	0.02 ± 0.00	0.02 ± 0.00	0.02 ± 0.00	0.02 ± 0.00
Ch	0.02 ± 0.01	0.04 ± 0.02	0.06 ± 0.04	0.07 ± 0.04	0.02 ± 0.01	0.04 ± 0.02	0.06 ± 0.04	0.07 ± 0.04
BbF	0.09 ± 0.01	0.12 ± 0.01	0.17 ± 0.03	0.20 ± 0.02	0.41 ± 0.04	0.45 ± 0.05	0.54 ± 0.09	0.63 ± 0.10
BkF	0.02 ± 0.00	0.02 ± 0.00	0.02 ± 0.00	0.02 ± 0.00	0.02 ± 0.00	0.02 ± 0.00	0.02 ± 0.00	0.02 ± 0.00
BaP	0.02 ± 0.00	0.02 ± 0.00	0.02 ± 0.00	0.02 ± 0.00	0.02 ± 0.00	0.02 ± 0.00	0.02 ± 0.00	0.02 ± 0.00
IP	0.02 ± 0.00	0.02 ± 0.00	0.02 ± 0.00	0.02 ± 0.00	0.02 ± 0.00	0.02 ± 0.00	0.02 ± 0.00	0.02 ± 0.00
DhA	0.02 ± 0.00	0.02 ± 0.00	0.02 ± 0.00	0.02 ± 0.00	0.02 ± 0.00	0.02 ± 0.00	0.02 ± 0.00	0.02 ± 0.00
BgP	0.02 ± 0.00	0.02 ± 0.00	0.03 ± 0.01	0.04 ± 0.01	0.02 ± 0.00	0.02 ± 0.00	0.04 ± 0.02	0.06 ± 0.02
total	3.27 ± 0.22	3.57 ± 0.29	4.05 ± 0.45	4.40 ± 0.34	5.62 ± 0.41	6.24 ± 0.43	7.48 ± 0.82	8.13 ± 1.02
PAH4	0.16 ± 0.01	0.20 ± 0.03	0.27 ± 0.07	0.32 ± 0.06	0.41 ± 0.12	0.45 ± 0.07	0.47 ± 0.09	0.55 ± 0.09

The means of PAHs compound based on the type of milk product, production date, and expiration are presented in Table 5. The results revealed that the means of all PAHs compounds were higher in expire date than the production date for the two tested milk products. In the expiration date, the maximum mean of both total PAHs and PAH4 were 7.38 ± 1.35 and 0.68 ± 0.15 μg/kg in butter and 4.11 ± 0.53 and 0.27 ± 0.09 μg/kg in yogurt. Additionally, the maximum mean of BaP in all fat status was below the standard limits of the EU. These compounds may be higher at the date of expiration for several reasons such as binding of these compounds together, turning from other substances to these compounds, and freeing them from containers (2020), but in general, the difference between production time and expiration time was not significant.

**TABLE 5:** Mean ± *SD* of PAH factors in yogurt and butter in Iran based on production date (μg/kg)

Type of dairy products				
Analyte	Yogurt		Butter	
	Production date			
	Production date	Expire date	Production date	Expire date
	Mean ± *SD*	Mean ± *SD*	Mean ± *SD*	Mean ± *SD*
NA	0.02 ± 0.00	0.02 ± 0.00	0.02 ± 0.00	0.02 ± 0.00
Ace	0.66 ± 0.26	0.95 ± 0.19	1.44 ± 0.22	1.79 ± 0.31
Ac	0.02 ± 0.00	0.02 ± 0.00	0.02 ± 0.00	0.02 ± 0.00
F	0.02 ± 0.00	0.02 ± 0.00	0.02 ± 0.00	0.02 ± 0.00
Pa	1.15 ± 0.07	1.27 ± 0.12	1.87 ± 0.25	2.15 ± 0.44
A	0.02 ± 0.00	0.02 ± 0.00	0.02 ± 0.00	0.02 ± 0.00
Fl	0.02 ± 0.00	0.02 ± 0.00	0.02 ± 0.00	0.02 ± 0.00
P	1.35 ± 0.06	1.44 ± 0.12	2.35 ± 0.30	2.56 ± 0.44
BaA	0.02 ± 0.00	0.02 ± 0.00	0.02 ± 0.00	0.02 ± 0.00
Ch	0.03 ± 0.01	0.07 ± 0.03	0.03 ± 0.01	0.07 ± 0.03
BbF	0.13 ± 0.04	0.16 ± 0.05	0.45 ± 0.06	0.57 ± 0.12
BkF	0.02 ± 0.00	0.02 ± 0.00	0.02 ± 0.00	0.02 ± 0.00
BaP	0.02 ± 0.00	0.02 ± 0.00	0.02 ± 0.00	0.02 ± 0.00
IP	0.02 ± 0.00	0.02 ± 0.00	0.02 ± 0.00	0.02 ± 0.00
DhA	0.02 ± 0.00	0.02 ± 0.00	0.02 ± 0.00	0.02 ± 0.00
BgP	0.02 ± 0.00	0.03 ± 0.01	0.02 ± 0.01	0.04 ± 0.02
total	3.54 ± 0.40	4.11 ± 0.53	6.35 ± 0.82	7.38 ± 1.35
PAH4	0.20 ± 0.05	0.27 ± 0.09	0.51 ± 0.07	0.68 ± 0.15

The means of PAHs analysts based on the type of brands are presented in Table 6 and indicated that the mean of all compounds in B and A brand in the samples showed the highest and lowest value, respectively. So that in brand B, the maximum mean of total PAHs and PAH4 were 7.22 ± 1.28 μg/kg and 0.62 ± 0.16 μg/kg in butter, and 3.91 ± 0.58 μg/kg and 0.25 ± 0.09 μg/kg in yogurt. On the other hand, the maximum mean of BaP in the tested brands was low compared with the standard levels of the EU. The possible reasons for this high level of PAHs in brand B may be due to the proximity of the produced animals to industrial environments, the pollution of water, air, or livestock feeds in this area.

**TABLE 6:** Mean ± *SD* of PAH factors in yogurt and butter in Iran based on brand type (μg/kg)

Type of dairy products						
Analyte	Yogurt			Butter		
	Brand type			Brand type		
	A	B	C	A	B	C
	Mean ± *SD*	Mean ± *SD*	Mean ± *SD*	Mean ± *SD*	Mean ± *SD*	Mean ± *SD*
NA	0.02 ± 0.00	0.02 ± 0.00	0.02 ± 0.00	0.02 ± 0.00	0.02 ± 0.00	0.02 ± 0.00
Ace	0.80 ± 0.28	0.85 ± 0.26	0.76 ± 0.29	1.63 ± 0.33	1.70 ± 0.33	1.53 ± 0.32
Ac	0.02 ± 0.00	0.02 ± 0.00	0.02 ± 0.00	0.02 ± 0.00	0.02 ± 0.00	0.02 ± 0.00
F	0.02 ± 0.00	0.02 ± 0.00	0.02 ± 0.00	0.02 ± 0.00	0.02 ± 0.00	0.02 ± 0.00
Pa	1.21 ± 0.12	1.22 ± 0.12	1.20 ± 0.12	2.04 ± 0.36	2.13 ± 0.39	1.86 ± 0.38
A	0.02 ± 0.00	0.02 ± 0.00	0.02 ± 0.00	0.02 ± 0.00	0.02 ± 0.00	0.02 ± 0.00
Fl	0.02 ± 0.00	0.02 ± 0.00	0.02 ± 0.00	0.02 ± 0.00	0.02 ± 0.00	0.02 ± 0.00
P	1.38 ± 0.10	1.40 ± 0.12	1.39 ± 0.09	2.45 ± 0.40	2.58 ± 0.40	2.34 ± 0.36
BaA	0.02 ± 0.00	0.02 ± 0.00	0.02 ± 0.00	0.02 ± 0.00	0.02 ± 0.00	0.02 ± 0.00
Ch	0.05 ± 0.03	0.05 ± 0.04	0.04 ± 0.03	0.05 ± 0.03	0.05 ± 0.04	0.04 ± 0.03
BbF	0.15 ± 0.05	0.16 ± 0.05	0.14 ± 0.05	0.51 ± 0.12	0.53 ± 0.12	0.49 ± 0.11
BkF	0.02 ± 0.00	0.02 ± 0.00	0.02 ± 0.00	0.02 ± 0.00	0.02 ± 0.00	0.02 ± 0.00
BaP	0.02 ± 0.00	0.02 ± 0.00	0.02 ± 0.00	0.02 ± 0.00	0.02 ± 0.00	0.02 ± 0.00
IP	0.02 ± 0.00	0.02 ± 0.00	0.02 ± 0.00	0.02 ± 0.00	0.02 ± 0.00	0.02 ± 0.00
DhA	0.02 ± 0.00	0.02 ± 0.00	0.02 ± 0.00	0.02 ± 0.00	0.02 ± 0.00	0.02 ± 0.00
BgP	0.03 ± 0.01	0.03 ± 0.01	0.02 ± 0.00	0.03 ± 0.02	0.04 ± 0.02	0.03 ± 0.02
Total	3.81 ± 0.57	3.91 ± 0.58	3.75 ± 0.54	6.90 ± 1.23	7.22 ± 1.28	6.49 ± 1.18
PAH4	0.23 ± 0.08	0.25 ± 0.09	0.22 ± 0.08	0.59 ± 0.15	0.62 ± 0.16	0.57 ± 0.14

### 3.3 Risk assessment of PAHs in milk products

**FIGURE 1:**
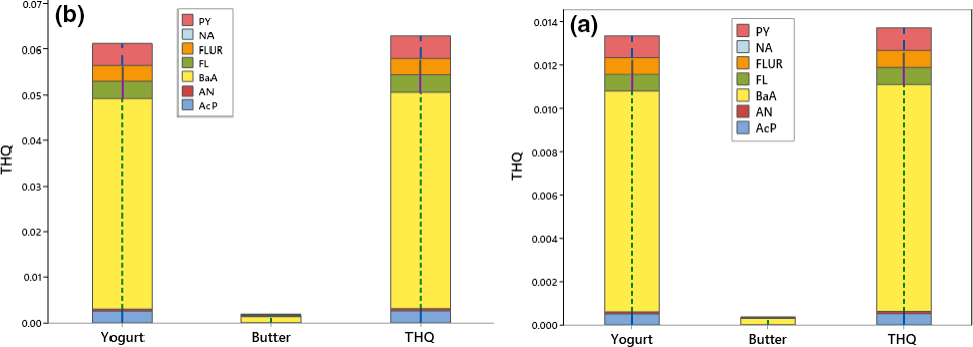
THQ value and percentage share due to content of 7 PAHs in the adult (a) and children (b) in milk products. PAHs, polycyclic aromatic hydrocarbons; THQ, total hazard quotient

**FIGURE 2:**
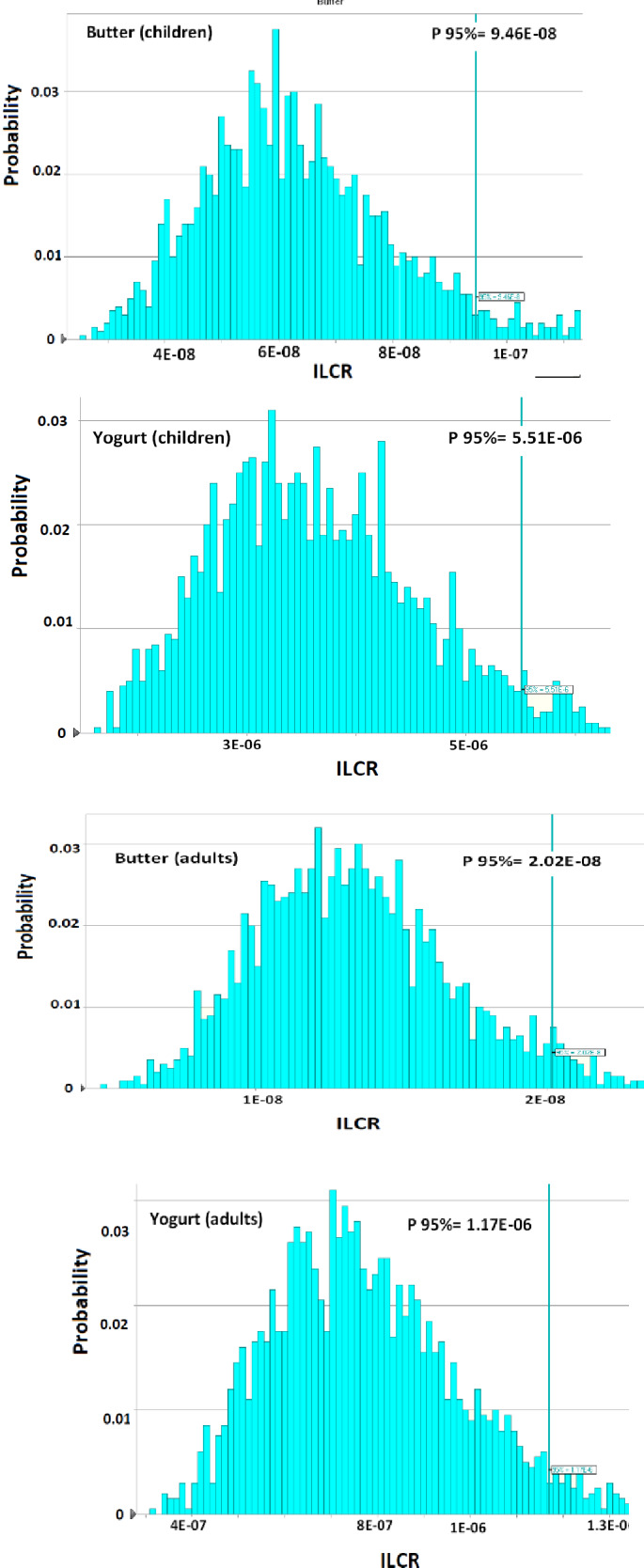
ILCR in the consumers due to ingestion yogurt and butter contents. ILCR, incremental lifetime of cancer risk

The toxicity equivalency quotient (TEQ) of benzo(a)pyrene was evaluated to assess the recommended health risk. Our outcome revealed that yogurts had the highest TEQBaP (0.105 μg/kg), while butter had less TEQBaP (0.066 μg/kg) as shown in Table 7. In a previous study, the level of TEQ in milk was reported as 0.21 μg/kg by Yoon et al. (2007) who examined the risk assessment of analytes of PAHs in milk sold in Korea. The non‐carcinogenic risk due to consumption of 7 PAHs in yogurt and butter showed that the rank order of HQ for adults and children was BaA > PY> F > FL>AcP > A > NA (Table 8). Because of the low oral reference dose (0.0003 mg kg^‐1^ day^‐1^), HQ of BaA was higher than other PAHs in milk products and the recorded values in these products were lower than 1 suggesting that subjects are not considerable at carcinogenic risk. The percentile of 95% THQ of 7 PAHs in the adult owing to ingestion of yogurt and butter was measured as 1.33E‐02 and 3.69E‐04, respectively. Furthermore, percentile 95% THQ in the children owing to ingestion of yogurt and butter was determined as 6.12E‐02 and 1.75E‐03, respectively. Moreover, THQ for adults and children was equal to 1.39E‐01 and 6.37E‐01, respectively. The comparison between THQ of PAHs in the samples showed that no carcinogenic risk due to the consumption of milk products for the adult and children since the recorded THQ of PAHs values were lower than 1. The share of the parameter in health risk of noncarcinogenic in the consumers due to ingestion of yogurt and butter obtained by the MCS (Figure 1a,b) concluded that BaA with 76.3% and 75.8% have a higher effect on THQ act in the adult and children, respectively. The results of carcinogenic risk of PAHs content due to the consumption of milk products (Figure 2) showed that ILCR index in adults owing to the consumption of yogurt and butter were 2.20E‐07 and 5.98E‐09, respectively. However, the percentile ILCR index in the children owing to ingestion of yogurt and butter were 1.02E‐06 and 2.89E‐8, respectively (Figure 2). Percentile 95% ILCR, PAHs in the yogurt and butter was between 1E‐4 to 1E‐9, hence, the carcinogenic risk for consumers is negligible (Ahmadkhaniha et al., 2009; USEPA, 2016). Furthermore, ILCR act in the adult and children was equal to 2.53E‐07 and 1.18E‐06, respectively; hence, the consumers are not at carcinogenic risk owing to the consumption of these milk products.

**TABLE 7:** Toxic equivalency factor (TEFs) and toxicity equivalency quotient TEQ BaP (μg/kg) concentrations in yogurt and butter

TEQBaP (μg/kg)			
Analytes	TEFs	Yogurt	Butter
NA	0.00100	0.00002	0.00002
Ace	0.00100	0.00162	0.00080
Ac	0.00100	0.00002	0.00002
F	0.00100	0.00002	0.00002
Pa	0.00100	0.00201	0.00121
A	0.01000	0.00020	0.00020
Fl	0.00100	0.00002	0.00002
P	0.00100	0.00245	0.00139
BaA	0.10000	0.00200	0.00200
Ch	0.01000	0.00046	0.00046
BbF	0.10000	0.05075	0.01463
BkF	0.10000	0.00200	0.00200
BaP	1.00000	0.02167	0.02167
IP	0.10000	0.00200	0.00200
DhA	1.00000	0.02000	0.02000
BgP	0.01000	0.00033	0.00025

**TABLE 8:** The percentile of 95% HQ due to ingestion of PAHs yogurt and butter

Analyte	RfD	Adults		Children	
		Yogurt	Butter	Yogurt	Butter
NA	0.02	5.18E−07	1.43E−08	2.43E−06	6.66E−08
AcP	0.06	5.05E−04	1.42E−05	2.44E−03	6.59E−05
FL	0.04	7.66E−04	2.08E−05	3.65E−03	9.95E−05
AN	0.30	1.02E−04	2.84E−06	4.66E−04	1.29E−05
FLUR	0.04	7.67E−04	2.08E−05	3.57E−03	9.98E−05
PY	0.03	1.01E−03	2.76E−05	4.77E−03	1.34E−04
BaA	0.0003	1.02E−02	2.83E−04	4.63E−02	1.33E−03
Total	0.00	1.33E−02	3.69E−04	6.12E−02	1.75E−03

## 4 CONCLUSIONS

This research provides a reliable, useful, and sensitive MSPE/GC‐MS technique for measurement and risk assessment of PAHs in yogurt and butter samples by using a simple technique for the preparation of samples. The results of our study showed that the BaP in all samples was below the EU standard level for milk products. The mean of total PAHs in butter samples was higher than yogurt samples. Based on HQ no public health risk was detected and ILCR revealed no significant potential for carcinogenic risk in both children and adult consumers.

The analytical methods used in the current study are accurate and practical. It is recommended to be used on the industrial scale to the checking of PAHs in other milk products, such as cream, ice cream, cheese, etc. for toxicological and epidemiological studies. Moreover, it will help to ensure the safety of dairy industries.

## CONFLICT OF INTEREST

The authors are fully aware that there is no competing interest in this study.

## ETHICAL REVIEW

This study does not involve any human or animal testing.

## Data Availability

The required data will be available upon request.
